# Efficacy and safety of Qi-Wei-Qing-Yan aerosol in treatment of acute pharyngitis (lung-stomach excess-heat syndrome): study protocol for a randomized controlled trial

**DOI:** 10.1186/s13063-016-1217-4

**Published:** 2016-02-19

**Authors:** Hong-li Jiang, Bin She, Wei Liu, Bing Mao, Ju-ying Zhang

**Affiliations:** Department of Integrated Traditional Chinese and Western Medicine, West China Hospital, Sichuan University, No. 37 Guoxue Street, Chengdu, 610041 Sichuan Province China; Department of Epidemiology and Biostatistics, School of Public Health, Sichuan University, No. 16 People’s South Road, Chengdu, 610041 Sichuan Province China

**Keywords:** acute pharyngitis, Qi-Wei-Qing-Yan aerosol, randomized controlled trial, sore throat, traditional Chinese medicine

## Abstract

**Background:**

Acute pharyngitis accounts for an estimated 15 million patient visits in the United States. However, there is no proven effective and safe treatment. Although Chinese herbal medicine is widely used in the treatment of acute pharyngitis, there is a lack of evidence-based data. Despite several clinical trials conducted in this setting, no randomized placebo-controlled trial has been performed to date. This trial aims to investigate the efficacy and safety of Qi-Wei-Qing-Yan aerosol (QWQYA), a Chinese herbal prescription, compared with a placebo aerosol in the treatment of acute pharyngitis with lung-stomach excess-heat syndrome.

**Methods/design:**

This is a prospective, multicenter, randomized, double-blinded, parallel-group, placebo-controlled trial. A total of 420 adult patients, of either sex, with acute pharyngitis will be enrolled from seven study sites across China. All patients will be randomly allocated to one of three parallel treatment groups: (1) QWQYA with the current propellant, (2) QWQYA with a previous propellant, and (3) the placebo aerosol with the current propellant. The study medication will be administered into the pharyngeal region in three sprays thrice daily for 5 consecutive days. The primary outcome measures are time to complete resolution of sore throat and relief rate of sore throat. Secondary outcome measures include resolution rate of sore throat, time to relief of sore throat, intensity of sore throat, and change of traditional Chinese medicine syndrome score and clinical signs score from baseline to post-treatment, as well as the occurrence of any adverse events.

**Discussion:**

This will be the first clinical trial to investigate the efficacy and safety of QWQYA in the treatment of acute pharyngitis in an adult population in a multicenter, randomized, double-blinded, parallel-group, placebo-controlled manner. Not only might it establish the basis for the efficacy and safety of QWQYA in treating acute pharyngitis, but it might also provide evidence to support the use of Chinese herbal medicine in treating acute pharyngitis and thus support an alternative treatment option for management of acute pharyngitis.

**Trial registration:**

Chinese Clinical Trial Registry ChiCTR-IPR-15005991.

## Background

Acute pharyngitis is characterized by an inflammation of mucous membranes of the pharynx and the surrounding lymphoid tissue and accounts for an estimated 15 million patient visits in 2006 in the United States [1]. It is commonly caused by various viral or bacterial infections. Viruses are the most common cause, accounting for up to 40 % to 60 % of cases [2]. The most common type of bacterial infection is group A beta-hemolytic streptococcal (GABHS) infection, which is responsible for around one-third of pediatric pharyngitis but only 5 % to 10 % of adult pharyngitis [3, 4]. An acute onset of sore throat is the predominant symptom, accounting for more than 10 % of all primary care visits [1].

Antibiotics are indicated to prevent suppurative complications and rheumatic fever, and decrease infectivity, but may only provide limited benefit in the resolution of symptoms, shortening sore throat duration by less than 1 day [5]. Moreover, excessive or inappropriate use of antibiotics may be associated with the potential side effects, increased antibacterial drug resistance, and high medical costs [6, 7]. Given that the majority of cases are caused by viral pathogens, the current treatment strategy in adults focuses on the relief of symptoms, particularly sore throat. Corticosteroids may reduce the severity and duration of pain, but corticosteroids alone should not be administered as routine treatment, owing to potential side effects [8–10]. Nonsteroidal anti-inflammatory drugs can reduce sore throat pain in 24 hours but are associated with gastrointestinal, renal, or hepatic adverse effects [11]. Although gargles or lozenges with local anaesthetic also provide sore throat pain relief, the effect is short-lived or temporary [3, 12].

Owing to a lack of proven effective and safe therapies in Western medicine, an increasing number of patients turn to complementary and alternative medicine [13–15], including traditional Chinese medicine, for relief of symptoms of acute pharyngitis. In traditional Chinese medicinal theory, acute pharyngitis falls into the disease category of pharyngitis (houbi in Chinese Pinyin), and is mainly caused by external causes, including wind and heat. Based on traditional Chinese medicinal clinical practice, wind-heat syndrome and lung-stomach excess-heat syndrome are the most common syndromes. Of these, lung-stomach excess-heat syndrome is more common in patients, with underlying heat in the lungs and stomach, and is primarily characterized by sore throat, cough, dryness of throat, thirst, and fever. The general traditional Chinese medicinal therapeutic principle is to clear away heat and toxins, and to relieve the sore throat.

Although traditional Chinese medicine has been widely used in China for the treatment of acute pharyngitis for thousands of years, Chinese herbal medicine is not recommended for use as an effective therapy, owing to the lack of high-quality clinical trials in this area [16]. Qi-Wei-Qing-Yan aerosol (QWQYA) is a registered herbal prescription in China and manufactured by Shandong Bencao Pharmaceutical Co., Ltd (Shandong, China). Table 1 lists the detailed formula. Despite the use of QWQYA in acute pharyngitis for several years, its efficacy has never been investigated through a placebo-controlled trial. Moreover, dichlorodifluoromethane (CFC-12), the previous propellant of QWQYA, has gradually been banned by the Ministry of Environmental Protection of the People’s Republic of China, owing to its ozone-depleting properties [17]. Alternatively, 1,1,1,2-tetrafluoroethane (HFC-134a) is used as the current aerosol propellant, owing to its negligible ozone depletion potential [18]. Accordingly, the safety of QWQYA with the current aerosol propellant remains unknown.

**Table 1:** Formula of Qi-Wei-Qing-Yan aerosol

Pinyin name	Botanical or zoological authority	Family name
Shandougen	*Euchresta japonica Hook. f. ex Regel*	Leguminosae
Shegan	*Belamcanda chinensis (L.) Redouté*	Iridaceae
Xuanshen	*Scrophularia ningpoensis Hemsl.*	Scrophulariaceae
Maidong	*Ophiopogon japonicus (L. f.) Ker-Gawl.*	Liliaceae
Chansu	*Bufo bufo gargarizans Cantor*	Bufonidae
Rengong Shexiang	*Moschus (Artificial)*	Not applicable
Bingpian	*Dryobalanops aromatica Gaertn.f.*	Dipterocarpaceae

The objective of this trial is to evaluate the efficacy and safety of two QWQYAs (active medication with the current or previous propellant) in comparison with a placebo aerosol (placebo with the current propellant) in patients with acute pharyngitis (lung-stomach excess-heat syndrome). In this study, the investigators hypothesize that (1) time to complete resolution of sore throat will be shorter in the groups using QWQYA with the current or previous propellant; (2) the proportion of patients who achieve sore throat pain relief will be higher in the groups using QWQYAs; and (3) QWQYA with the current propellant will be safe.

## Methods/design

**Fig. 1:**
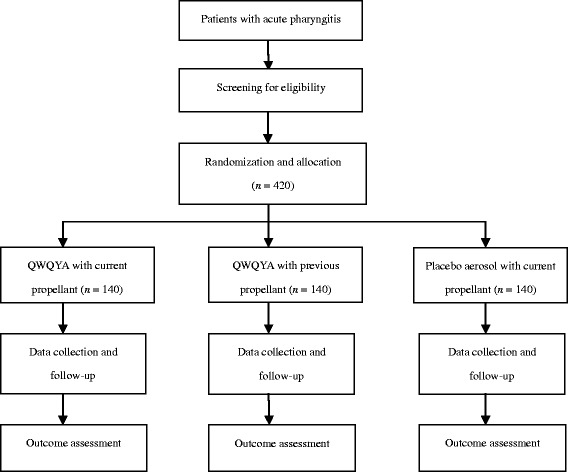
Study flow chart. *QWQYA* Qi-Wei-Qing-Yan aerosol

This is a prospective, multicenter, randomized, double-blinded, parallel-group, placebo-controlled trial of two QWQYAs (active medication with the current or previous propellant) versus placebo aerosol (placebo with the current propellant) for acute pharyngitis. The study has been authorized by the China State Food and Drug Administration (Approval No. 2014 L00188). In addition, the study was registered with the Chinese Clinical Trial Registry (ChiCTR-IPR-15005991). The trial protocol is conducted in accordance with the Declaration of Helsinki, the code of Good Clinical Practice, and the guidelines of the International Conference on Harmonisation. Recruitment is scheduled to occur from early April 2015 until October 2015. Figure 1 shows the flow chart of the trial.

### Patient population and setting

The diagnosis is made by medically qualified physician based on the presence of acute onset symptoms and signs suggestive of acute pharyngitis [19]. All patients will be screened for Centor criteria (history of fever (>38 °C), tonsillar exudates, tender anterior cervical adenopathy, and lack of cough) to focus on viral acute pharyngitis. Adults with none or only one of these findings will be included. Traditional Chinese medicinal diagnosis of lung-stomach excess-heat syndrome is based on the Guidelines for Clinical Research of New Chinese Medicine [20]; detailed criteria are presented in Table 2.

**Table 2:** Traditional Chinese medicine syndrome diagnostic criteria for lung-stomach excess-heat syndrome

Category	Symptoms or signs
Main symptom	Sore throat
Minor symptoms	Cough, dryness of throat, thirst, fever, dry stool, deep-colored urine
Tongue appearance	Light red appearance
Tongue coating	Thin yellow coating
Pulse	Rapid and powerful pulse

The syndrome can be diagnosed by the presence of the main symptom and ≥3 minor symptoms.

A written, signed, and dated informed consent form should be obtained from each patient before any study specific procedures are performed. The participant information sheet and informed consent form will be presented to patients by the responsible clinician, who will explain the exact nature of the study, the implications and constraints of the protocol, the known side effects, and any benefits or risks involved in participation, and the importance of completing the study. It is clearly stated that the participant is free to withdraw from the study at any time for any reason without prejudice to future care, and with no obligation to give the reason for withdrawal.

A total of 420 patients of either sex, aged 18–65 years, will be enrolled from seven study sites across China: (1) West China Hospital of Sichuan University, (2) Xiyuan Hospital of China Academy of Chinese Medical Sciences, (3) the first Affiliated Hospital of Guiyang College of Traditional Chinese Medicine, (4) Ruikang Hospital Affiliated to Guangxi University of Chinese Medicine, (5) the Affiliated Hospital of Shanghai University of Traditional Chinese Medicine, (6) the Affiliated Hospital to Liaoning University of Traditional Chinese Medicine, and (7) the Affiliated Hospital of Chengdu University of Traditional Chinese Medicine. A research assistant in each center will recruit patients either from outpatient departments, or through advertisements in community hospitals or pharmacies. Each participating center will recruit 60 patients.

#### Inclusion criteria

1. Clinical diagnosis of acute pharyngitis;
2. Lung-stomach excess-heat syndrome in traditional Chinese medicine;
3. Within 48 hours of onset;
4. Centor criteria ≤1;
5. Baseline sore throat score by visual analog scale of 5 or more, and by traditional Chinese medicine score of 6 or more;
6. Aged 18 to 65 years;
7. Patient must voluntarily give written, informed consent and report adverse events and concomitant medication for duration of study.

#### Exclusion criteria

1. Patients with temperature greater than 38.5 °C;
2. Use of any medication taken for relief of sore throat prior to study initiation;
3. Current use of analgesic or anti-inflammatory regimen, such as analgesics, nonsteroidal anti-inflammatory drugs, or steroids;
4. Chronic pharyngitis;
5. Patients with any suspected or known heart disease;
6. Sore throat caused by local irritation of mucous membranes due to gastroesophageal reflux or ingestion of caustic substances;
7. Patient with severe primary disease of pulmonary, hepatic, renal, or hematological system, or other serious diseases affecting survival, such as cancer or AIDS;
8. Abnormal results to myocardial markers test, electrocardiogram abnormality, alanine transaminase or aspartate aminotransferase > 1.5 times of normal upper limit, abnormality of serum creatine, white blood cell count < 3 × 10^9^/l or >10 × 10^9^/l, and neutrophil granulocyte >80 %;
9. Pregnancy or potential pregnancy or lactation;
10. Allergic constitution or known allergy to any component in QWQYA;
11. Patients taking similar medicines in the previous month or having participated or participating in other trials in previous 3 months;
12. Mentally or legally disabled patients.

### Withdrawal criteria

Once randomly allocated to a group, a patient will be regarded as included in the study. However, patients may voluntarily withdraw from the study at any time for lack of efficacy, worsening condition, or any other reason. Researchers can withdraw patients from the trial owing to patients’ lost to follow-up, development of allergic reaction, serious adverse events, condition apparently worsening, use of forbidden medications or treatments, unblinding, or actual usage of trial medication of <80 % or >120 % of the required dosage. Withdrawn patients will not be replaced. Any participant will be perceived as having completed the study if all post-treatment assessments have been performed.

### Termination criteria

The entire study will be terminated prematurely or suspended by the investigators in the case of serious adverse events, poor efficacy observed during the study, or significant deviation from the protocol, by the National Pharmaceutical Supervisory and Administrative Department for any reason, or by the sponsor due to management or funding problems.

### Randomization and blinding

Stratified blocked randomization will be applied in the study. Randomization sequence is generated in blocks of seven in a 1:1:1 ratio by an independent statistician not involved with the participants’ enrollment or study analysis, using Statistical Analysis System SAS (SAS 9.3). The random treatment assignment for each patient will be concealed and stored in advance in two opaque, sealed, sequentially numbered envelopes. Each set of envelopes corresponds to a unique allocation code containing the specified treatment group. The sealed envelopes are kept in a fireproof place, and will only be opened in case of a medical emergency. Participants, investigators, coordinators together with statisticians are all blinded to group assignments throughout the study.

### Investigational medicinal product

Both QWQYAs contain 6.2 g/7 ml of the active solution with the excipients of ethanol, propylene glycol, and span-80 (one with the current propellant and the other with the previous propellant). Placebo aerosol with the current propellant contains 0.31 g/7 ml of placebo solution with the same excipients for appropriate blinding. Each spray equals 32.6 mg/0.037 ml of active solution and 1.6 mg/0.037 ml of placebo solution.

**Fig. 2:**
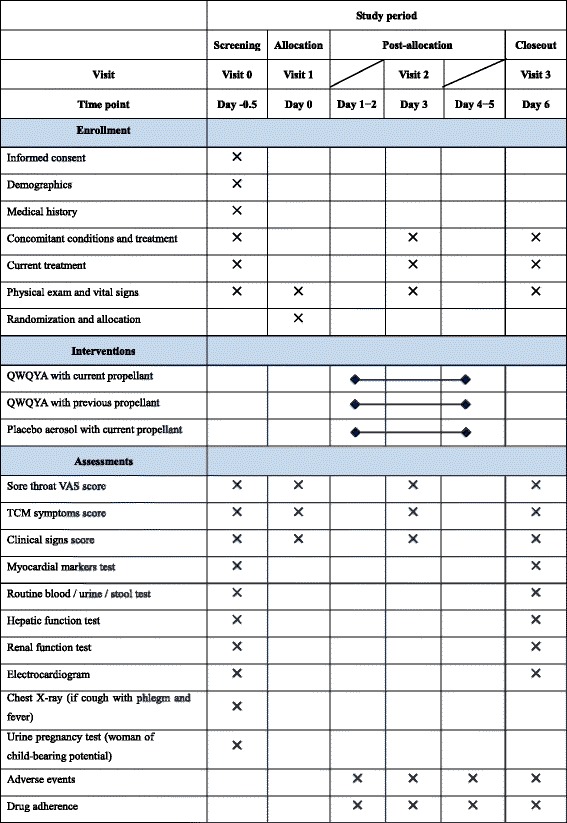
Schedule of study procedures. Participants will receive the study medication for 5 days after enrollment and allocation. The time-points of assessments are shown in the schedule. QWQYA, Qi-Wei-Qing-Yan Aerosol; TCM, traditional Chinese medicine; VAS, visual analog scale

Both active and placebo aerosols are manufactured by Shandong Bencao Pharmaceutical Co., Ltd., in accordance with the principles of Good Manufacturing Practice. Medication supplies are coded, packed, and labelled in a blinding manner according to the randomization list. Each investigational medicinal product package contains an aerosol. Both active and placebo aerosols are issued in bottles identical in shape, size, and color. A clearly visible label on each package states ‘FOR TRIAL USE ONLY’ and other information, including function and indication, name, dose, dosing schedule, storage condition, expiry date, and the manufacturer’s name. An independent drug administrator in each center is responsible for receipt, handling, storage, dispensing, and retrieval of all investigational medicinal products. The standard operating procedures, and the manner in which the records are kept, must be documented. An overview of consent, screening, enrollment, intervention, timing of measurements, and data collection is shown in Fig. 2.

### Interventions

All participants are randomly allocated to one of three parallel treatment groups: (1) QWQYA with the current propellant, three sprays into the pharyngeal region thrice daily; (2) QWQYA with the previous propellant, three sprays into the pharyngeal region thrice daily; and (3) the placebo aerosol, three sprays into the pharyngeal region thrice daily. The scheduled treatment duration will be 5 consecutive days.

### Concomitant treatments and forbidden medication

Patients can take any Western medication or other Chinese herbal medicine that may relieve sore throat or other symptoms of acute pharyngitis, or use antibiotics and anti-viral agents during the study, but then they will be withdrawn from the study. Medications used to control underlying conditions, such as hypertension or diabetic mellitus, are allowed. Any concomitant treatment or medication administered must be recorded carefully in case report forms, including the name, dose, dosing schedule, mode of administration, and treatment period.

### Drug adherence and compliance

Drug adherence is expected to be good, owing to the short study duration of 5 days and three visits after recruitment. Measures taken to maximize adherence include careful screening, fully informing patients of the potential benefits and risks, and attentive follow-up. Compliance will be checked by patients’ documented daily diaries, reporting how many sprays and times they apply the study medication, and by weighing each dispensed aerosol on the date on which the study medication is issued, and again on the date on which it is returned by the patient.

### Outcome measures

#### Primary outcome measures

1. Time to complete resolution of sore throat, i.e., length of time from the start of treatment until complete resolution. Sore throat will be assessed using an 11-point (0 to 10) numeric visual analog scale, with 0 indicating ‘no pain’ and 10 indicating ‘worst possible pain’. This score will be evaluated thrice daily, immediately before spraying. Complete resolution of sore throat is defined as a reduction of the score to 1 or less, lasting for more than 24 hours [21].
2. Relief rate of sore throat: this is defined as a reduction by 50 % in the baseline visual analog scale score recorded prior to treatment (day 0), lasting for more than 24 hours. The relief rate of sore throat is the proportion of patients who achieve relief of sore throat.

#### Secondary outcome measures

1. Resolution rate of sore throat: proportion of patients who achieve complete resolution of sore throat.
2. Time to relief of sore throat: length of time from the start of treatment until pain relief.
3. Change of intensity of sore throat: assessed by visual analog scale from baseline to post-treatment.
4. Change of traditional Chinese medicine syndrome score from baseline to post-treatment: each traditional Chinese medicine symptom is graded (Fig. 3), and the traditional Chinese medicine syndrome score is the accumulated score of the main symptom score and all minor symptom scores.

**Fig. 3:**
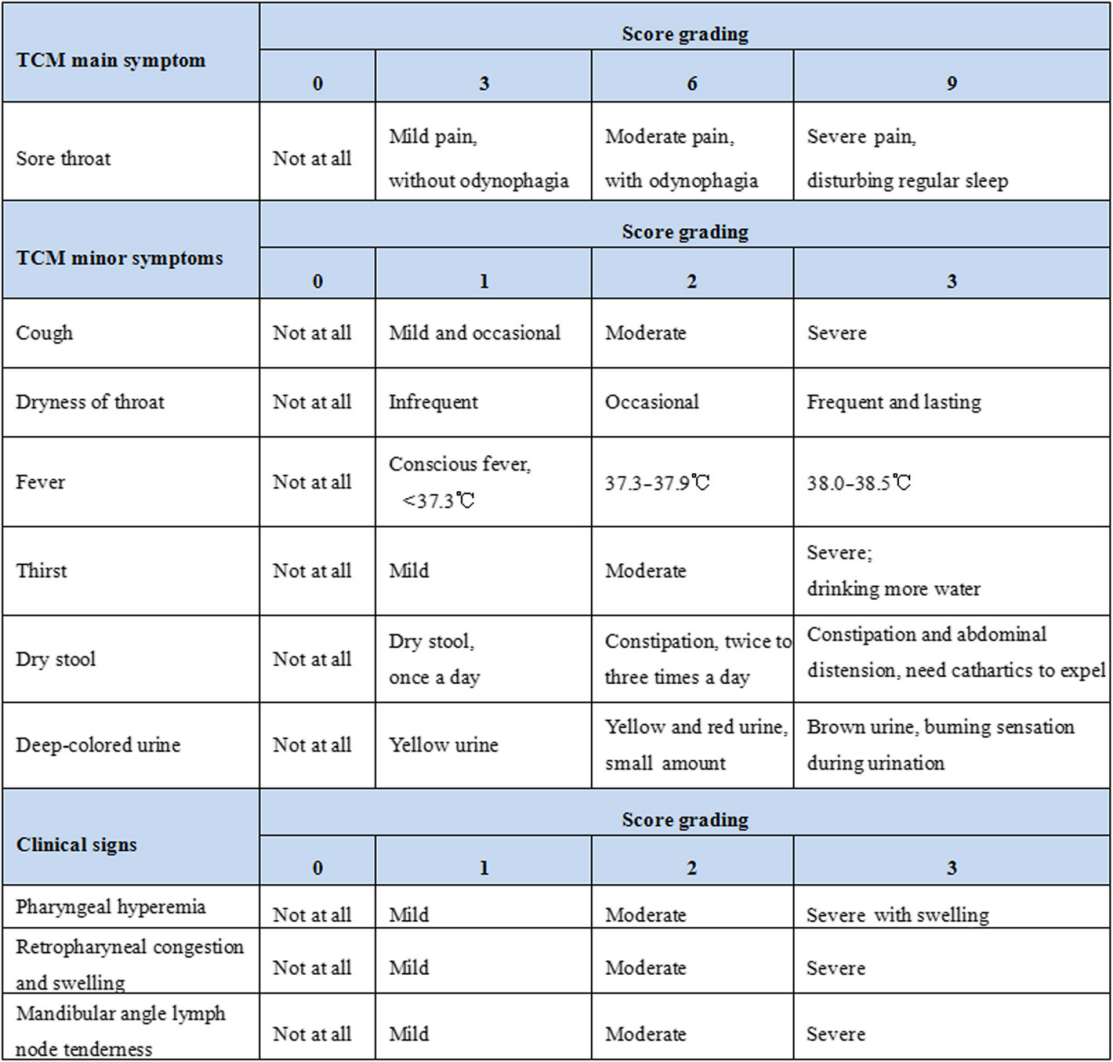
Symptoms and signs score. TCM, traditional Chinese medicine
5. Change of clinical signs score.

Three primary clinical signs in acute pharyngitis will be observed and recorded before and after treatment. The detailed scoring criteria are shown in Fig. 3.

### Safety outcome measures

Safety outcome measures include monitoring of adverse events, as well as clinical and laboratory findings. In addition to routine tests of blood, urine, and stool, hepatic and renal functions, and myocardial markers, electrocardiography will also be performed at baseline and again after treatment, to assess safety for each group. Patients will be required to record any unexpected symptoms or signs during the treatment period.

### Data management, monitoring, and auditing

A paper case report form will be used to collect data. To ensure maximum follow-up evaluation, the following measures will be taken:

1. Train investigators and study staff in the importance of keeping participants in the trial until the end.
2. Ensure that participants completely understand the research objectives and importance of completing the study.
3. Keep contact information for participants including phone numbers, email addresses, and mailing addresses.
4. Remind participants of their appointments.
5. Make follow-up appointments at each participant’s convenience.
6. Provide a remuneration of Chinese ¥200 after completing the study.
7. Provide continued access to effective treatments after the trial for patients whose symptoms are not yet cured.
8. Make efforts to obtain the participant’s consent for the collection of data outcomes when medication discontinuation occurs.

After completion of data collection, double data entry will be performed to transcribe the data into the computer database as soon as possible after recruitment and follow-up evaluation so that the patient and data collector are still available if responses are found to be missing or out of range. All information on the patients and outcome variables will be stored, updated, and monitored using EpiData3.1, which will also be used to format the data for statistical analysis. Regular backups and off-site storage will be conducted, to prevent loss of the database. The principal investigator, data managers, sponsor, and statisticians will confirm and lock the database prior to data processing. Only data managers and statisticians will have access to the final data. Original case report forms and any other records will be kept on file at each study site for 5 years.

Interim analyses will not be performed, as the investigational product is a registered and clinically used aerosol and the study is scheduled to last for approximately 6 months. An independent data monitoring committee will be set up to monitor patient safety and treatment efficacy data. Moreover, a monthly progress report on enrollment, recruitment, study participations, safety, and completion will be issued to the principal investigator. The regulatory authority at each study site and auditors independent of the investigators and sponsor will conduct a monthly audit, including, but not limited to, trial procedure and compliance with the protocol, standard operating procedures, code of Good Clinical Practice, and the applicable regulatory requirements. Any observation or finding should be documented.

### Adverse event reporting

An adverse event will be defined as any unfavorable and unintended sign (including abnormal laboratory findings), symptom, or disease temporally associated with the use of the study medication. All adverse events will be recorded in detail, including event, date of occurrence, grade, whether expected or unexpected, date resolved, and treatment patients receive specifically related to the event, and will be categorized as clearly not, doubtfully, may be, likely, or clearly related to the study medication. A serious adverse event will be defined as any untoward medical occurrence that, at any dose, results in death, is life-threatening, requires inpatient hospitalization or prolongation of existing hospitalization, results in persistent or significant disability or incapacity, is a congenital anomaly or birth defect, or other important medical event. Other events that are not likely to result in death, are not life-threatening, or do not require hospitalization, might be considered as serious adverse event if the event might jeopardize the patient and might require medical or surgical intervention to prevent one of the outcomes listed above.

Medically qualified investigators will record, assess, and report adverse events. Non-serious adverse events will be recorded in case report forms, and listed in a monthly progress report to the principal investigator and sponsor. Serious adverse events must be reported to the institutional review board, principal investigator, sponsor, and the Drug Registration Division of China’s State Food and Drug Administration and Research within 24 hours. The blinding procedures will be broken in case of a medical emergency.

### Sample size calculation

The sample size was calculated on the basis of the two primary outcomes, using ‘Non-inferiority tests for two proportions [differences]’ in PASS 11.0. Based on a previous study [22], the mean time to complete resolution of sore throat in both active treatments was similar, both with a standard deviation of 2.2 days. A difference of 1 day or more between groups was regarded a clinically important effect. Setting a one-sided *α* of 0.05 and *β* of 0.1, the sample size was estimated to be 84 cases for each group. Based on consultation with clinical experts and a preliminary unpublished study provided by the sponsor, the relief rate of sore throat in QWQYA with previous propellant was estimated at 91 %, with a difference of 10 % or more considered clinically significant. The sample size was estimated at 118 participants for each group, assuming a one-sided *α* of 0.05 and *β* of 0.15. Both the sample sizes were calculated according to the formula:

*n* = $$ {\left[\frac{2\left({Z}_{1-\alpha /2}+{Z}_{\beta}\right)\sigma }{\delta -\varDelta}\right]}^2 $$

Therefore, the final sample size for each group was 118 participants. Taking approximate dropout rates of 20 % into account, the number of participants to be recruited was estimated at 140 per group. Therefore, a total of 420 patients will be recruited in this trial.

### Statistical analysis

An independent statistician will perform statistical analysis using SAS 9.3. The full analysis set population includes all randomized participants as allocated. Only missing data regarding the primary outcomes will be adjusted using the last observation carried forward method. The per-protocol population includes only patients who fulfil the protocol. Analysis of efficacy will be performed based on the full analysis set and per-protocol population. Safety analyses will be performed in those populations, which include all randomized participants who receive at least one spray of medication and who have at least one visit for safety, whether or not they are withdrawn prematurely.

A fully specified statistical analysis plan will be written before unmasking. Quantitative variables will be described as mean ± standard deviation or median, and qualitative variables as frequency and percentage. Baseline analyses will be performed using analysis of variance (ANOVA) or rank-sum test for quantitative data and chi-squared or Fisher’s exact test for qualitative data. A non-inferiority test will be used to compare the two active QWQYA groups, and a superiority test to compare the current-propellant QWQYA group and the placebo group. A one-sided confidence interval method will be used in efficacy analyses. Statistical significance will be considered for *P* ≤ 0.05.

### Ethical considerations and dissemination

The study protocol has been reviewed and approved by the Biomedical Ethics Committee of West China Hospital of Sichuan University (Chengdu, China). Any modifications, including the principal investigator, study protocol, and informed consent forms must be re-submitted, reviewed, and approved by the Biomedical Ethics Committee. Trained research investigators will explain the study procedures using information sheets, which will be provided to all potential participants prior to participation. All participants must voluntarily give their written, informed consent prior to any study procedures. Participants with impaired decision-making capacity who might have difficulty weighing the risks and benefits of the study will be excluded from this study. The principal investigator, sponsor, ethical committees, and drug regulation agencies have access to individual patient’s data, and no ancillary studies will use these participants’ data. Moreover, each patient will be identified with a unique identity without any other personal identifier for purposes of maintaining confidentiality. This study protocol has been prepared according to the SPIRIT checklist [23].

Additionally, chest X-rays will be obtained in selecting eligible participants, to exclude patients with lower respiratory tract infection, and a urine pregnancy test (strip) will be used to detect early suspected or unknown pregnancy in women with child-bearing potential.

A preliminary study (an unpublished observation by Professor Jia Miao) suggests that QWQYA might be associated with a risk of mild reduction of heart rate within normal range. However, another two trials [24, 25] did not find any such association. In this study, myocardial markers will be tested and electrocardiography will be performed in potential participants, and a detailed history will be taken, to exclude those with an underlying heart disease. In addition, the test of myocardial markers and electrocardiography will be performed again after treatment to monitor participants for potential adverse effects.

The results of this study will be presented at national or international conferences and submitted to peer-reviewed journals.

## Discussion

A Cochrane review [16] of 12 randomized controlled trials that assessed the efficacy and safety of Chinese herbal medicine for sore throat found that trials in this area were methodologically of poor quality, with controversial and questionable evidence of efficacy. Moreover, the randomized controlled trials included in this review and trials conducted thereafter [26–28] all used a ‘positive effect drug’ as a control, comparing the Chinese herbal medicine under investigation with another Chinese herbal medicine or a Western medicine. However, the efficacy of these ‘positive effect drugs’ has not yet been well established. To the best of our knowledge, this will be the first multicenter, double-blind, randomized, placebo-controlled trial of Chinese herbal medicine designed to treat acute pharyngitis in an adult population. Not only might it establish the basis for the efficacy and safety of QWQYA in the treatment of acute pharyngitis, but it might also provide evidence regarding Chinese herbal medicine for treating acute pharyngitis and an alternative treatment option for the management of acute pharyngitis.

This trial is placebo-controlled and focuses on viral pharyngitis in adults. Although it is not always easy to distinguish viral from bacterial causes, laboratory confirmation of bacteria, including a rapid antigen detection test and throat culture is not indicated and recommended in adults with a lower likelihood of GABHS infection [29]. In this trial, Centor criteria (a history of fever, tonsillar exudates, tender anterior cervical adenopathy, and lack of cough) will be applied to exclude patients with potential GABHS pharyngitis, with the absence of three or four of the criteria indicating a negative predictive value of more than 80 % [30, 31].

It is well-known that the gold standard for assessing the efficacy of specific intervention is a randomized placebo-controlled trial. Ideally, a placebo should be an inactive substance that is pharmacodynamically inert. However, it is significantly difficult to develop appropriate placebos, in terms of smell, taste, and color, in clinical trials of herbal medicine especially those of compound formula in a liquid form. To our knowledge, no validated placebo of Chinese herbal medicine has been developed in the setting of acute pharyngitis. Moreover, several clinical trials outside the setting of acute pharyngitis have applied 10–20 times dilutions of active herbs as placebos in China and Japan [32, 33]. In this trial, one-twentieth of the total dose of active solution is used as the placebo, since it is assumed to be unlikely that a tiny amount of 14.4 mg per day could be an effective treatment. Both the active aerosols and the placebo aerosol are issued in bottles identical in size, shape, and color, to ensure appropriate blinding. In addition, participants are required not to communicate with each other, to minimize the possibility of revealing differences between the placebo and active medication.

### Trial status

This trial is preparing for patient recruitment at the time of manuscript submission.

## Abbreviations

ANOVA: analysis of variance
GABHS: Group A beta-hemolytic streptococcal
QWQYA: Qi-Wei-Qing-Yan aerosol
